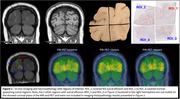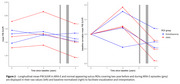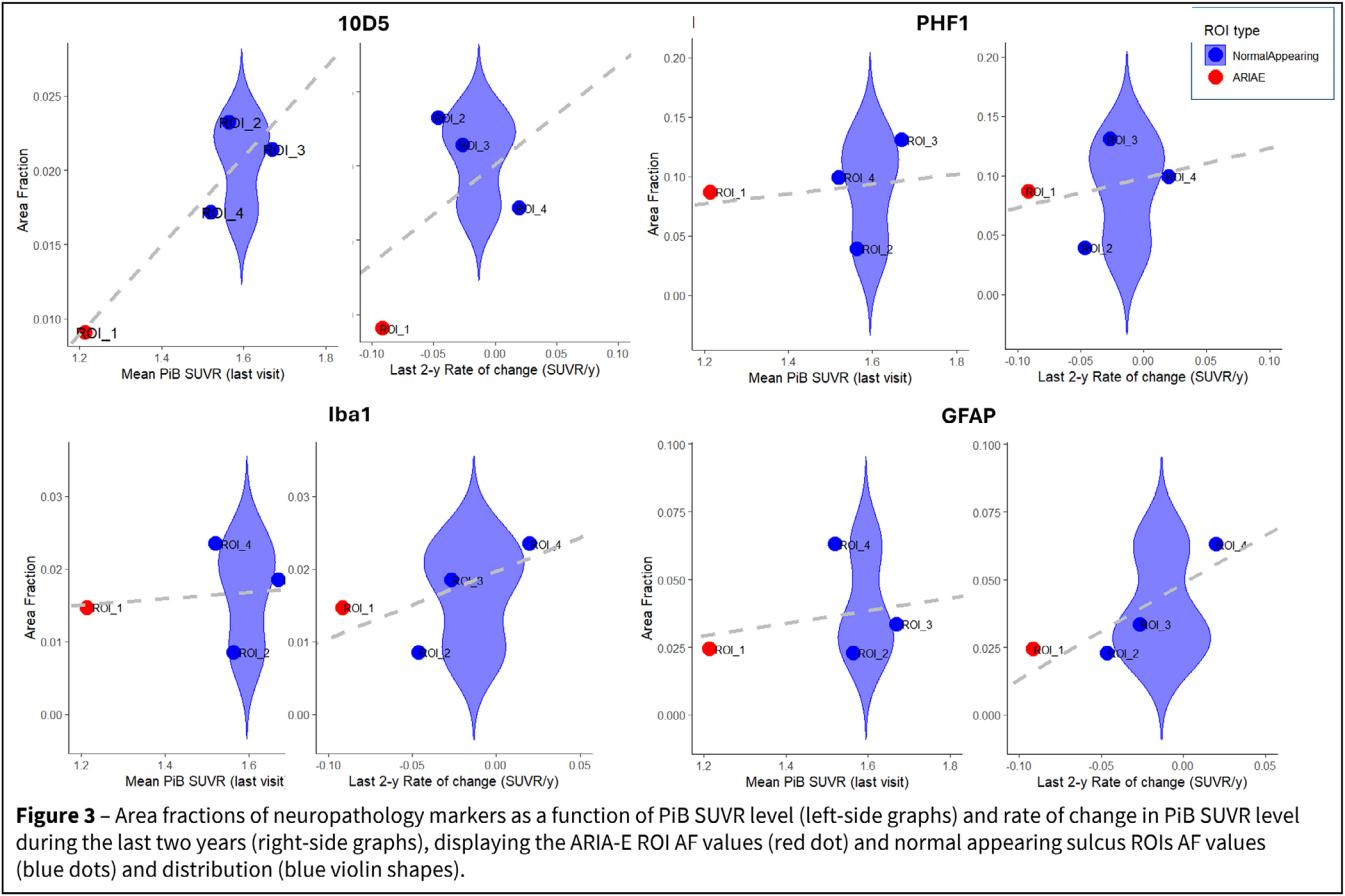# Imaging‐neuropathology evaluation of Amyloid Related Imaging Abnormality sulcal effusion in a DIAN‐TU‐001 participant treated with gantenerumab

**DOI:** 10.1002/alz70862_110062

**Published:** 2025-12-23

**Authors:** Nelly Joseph‐Mathurin, Charles D Chen, Diana A Hobbs, Meng Jiang, Shaney Flores, Jalen Scott, Sanjana Anand, Erin E. Franklin, Ruijin Lu, Brian A. Gordon, Clifford R. Jack, Carlos Cruchaga, Jason J. Hassenstab, Laura Ibanez, Yan Li, Guoqiao Wang, Alan E. Renton, Chengjie Xiong, Alireza Atri, B. Joy Snider, Jorge J. Llibre‐Guerra, David B. Clifford, Eric McDade, Randall J. Bateman, Qing Wang, Richard J. Perrin, Tammie L.S. Benzinger

**Affiliations:** ^1^ Washington University School of Medicine, St. Louis, MO USA; ^2^ Massachusetts General Hospital, Charlestown, MA USA; ^3^ George Washington University, Washington DC, DC USA; ^4^ Mayo Clinic, Rochester, MN USA; ^5^ Icahn School of Medicine at Mount Sinai, New York, NY USA; ^6^ Banner Sun Health Research Institute, Sun City, AZ USA; ^7^ Washington University School of Medicine in St. Louis, St. Louis, MO USA

## Abstract

**Background:**

Amyloid related imaging abnormality edema (ARIA‐E) occurs in about 19% of individuals with autosomal dominant Alzheimer disease (ADAD) treated with an anti‐amyloid‐b monoclonal antibody (Salloway et al., 2021). In a previously reported case, ARIA‐E appeared to colocalize with decreases in PiB‐PET uptake (Joseph‐Mathurin, Llibre‐Guerra, et al, 2022), suggesting an association between amyloid‐b (Aβ) removal and ARIA‐E. Here, we compare longitudinal neuroimaging and corresponding neuropathology in an ADAD individual treated with gantenerumab, who experienced multiple ARIA‐E episodes as sulcal effusions that resolved by the end of the four‐year trial.

**Methods:**

PiB‐PET and MRI were collected pre‐ and post‐randomization (2 and 4 years). The participant consented to brain donation, which was received a year after trial completion. Four tissue samples were taken from the location of the ARIA‐E in the parieto‐occipital left hemibrain as identified by MRI (one block contained the sulcus affected by ARIA‐E; the rest of the coronal section was captured in the remaining three blocks). Area fractions (AFs) of Aβ (10D5 immunohistochemistry (IHC)), tauopathy (PHF1), microglia (Iba1), and astrocytes (GFAP) in one sulcal region of interest (ROI) from each block and PiB‐PET SUVRs of corresponding ROIs were extracted (*n* = 4, Figure 1). Additionally, we included PiB‐PET SUVRs corresponding to ARIA‐E findings observed in the right hemisphere at baseline, two‐year, and four‐year visits (*n* = 6).

**Results:**

Overall PiB‐PET uptake increased during the first two years (before ARIA‐E) and decreased during the last two years (including ARIA‐E episodes) (0.03±0.05 vs. ‐0.04±0.04 SUVR/year, *p*‐value=0.01, Figure 2). The decrease seemed more pronounced in ARIA‐E ROIs versus normal‐appearing sulcus ROIs (‐0.05±0.04 vs. ‐0.02±0.03 SUVR/year). Lower PiB uptake at last visit appeared associated with lower Aβ AF (estimates=0.03±0.01, *p*‐value=0.07). The ARIA‐E ROI had an Aβ AF of 0.009 while normal‐appearing sulcus ROIs had a mean of 0.02+0.003 (Figure 3). AFs for tauopathy, microglia, and astrocytes were within the range of those of normal‐appearing sulcus ROIs, suggesting an Aβ‐specific effect, although unexamined markers may play a role.

**Conclusions:**

Our preliminary findings indicated that ARIA‐E is associated with longitudinal PiB‐PET decrease and Aβ AF, supporting the link between ARIA‐E, changes in PiB‐PET, and local Aβ removal observed at autopsy.

**Funding**: K01AG080123; U01AG042791; R01AG046179; R01AG053267